# The Influence of Adiposity Levels on the Relation between Perfluoroalkyl Substances and High Depressive Symptom Scores in Czech Adults

**DOI:** 10.3390/toxics11110946

**Published:** 2023-11-20

**Authors:** Geraldo Neto, Martin Bobak, Juan P. Gonzalez-Rivas, Jana Klanova

**Affiliations:** 1 International Clinical Research Center (ICRC), St. Anne’s University Hospital (FNUSA), 65691 Brno, Czech Republic; juan.gonzalez@fnusa.cz; 2 Research Centre for Toxic Compounds in the Environment (RECETOX), Faculty of Science, Masaryk University, 62500 Brno, Czech Republic; martin.bobak@recetox.muni.cz (M.B.); jana.klanova@recetox.muni.cz (J.K.); 3 Research Department of Epidemiology and Public Health, University College London, London WC1H 9BT, UK; 4 Department of Global Health and Population, Harvard TH Chan School of Public Health, Harvard University, Boston, MA 02138, USA; 5 Foundation for Clinic, Public Health, and Epidemiology Research of Venezuela (FISPEVEN INC), Caracas 3001, Venezuela

**Keywords:** perfluoroalkyl substances, PFOA, PFDA, PFNA, PFOS, depression, body fat, adults

## Abstract

The extensive use and bioaccumulation of Perfluoroalkyl Substances (PFAS) over time raise concerns about their impact on health, including mental issues such as depression. This study aims to evaluate the association between PFAS and depression. In addition, considering the importance of PFAS as an endocrine disruptor and in adipogenesis, the analyses will also be stratified by body fat status. A cross-sectional study with 479 subjects (56.4% women, 25–89 years) was conducted. Four PFAS were measured: perfluorooctanoic acid (PFOA), perfluorononanoic acid (PFNA), perfluorodecanoic acid (PFDA), and perfluorooctane sulfonate (PFOS). The Poisson regression model was applied using robust error variances. The fully adjusted model included age, sex, educational level, income, smoking, physical activity, body fat percentage, and the questionnaire to assess depression. The prevalence of depression and high body fat was 7.9% and 41.1%, respectively. Only PFOA was significantly associated with depression in the entire sample (prevalence rate (PR): 1.91; confidence interval (CI95%): 1.01–3.65). However, in the group with normal adiposity, PFOA (3.20, CI95%: 1.46–7.01), PFNA (2.54, CI95%: 1.29–5.00), and PFDA (2.09, CI95%: 1.09–4.00) were also significant. Future research should investigate the role of obesity as well as the biological plausibility and possible mechanisms increasing the limited number of evidences between PFAS and depression.

## 1. Introduction

For a period in excess of eight decades, per- and polyfluoroalkyl substances (PFAS) have undergone extensive synthesis and application in a myriad of commercial and industrial contexts [1]. These synthetic chemicals can be found in everyday items such as electronics, automotive supplies, and food packaging. Additionally, they can be present in non-stick cookware, stain- and water-resistant coatings, cleaning products, waxes, and fire-fighting foams [2]. Their exceptional durability and resistance to decomposition render PFAS compounds persistently impactful on the environment, thereby posing potential long-term ramifications for human health. The ongoing and widespread exposure to PFAS is principally facilitated through diverse pathways, namely, ingestion via drinking water and dietary intake; inhalation of outdoor air; and contact with indoor dust and soil [3,4]. Consequently, PFAS exposure predominantly occurs through the intricate interplay of ingestion and absorption into the bloodstream via the gastrointestinal tract, though alternative routes such as inhalation and dermal absorption also contribute significantly [5]. The structural underpinning of PFAS lies in a succinct yet robust carbon-fluorine bond, endowing these compounds with chemical stability and remarkable resistance to organic degradation. This structural feature imparts bioaccumulation tendencies to substances containing PFAS, as they persist and accumulate over temporal scales [6]. The presence of PFAS in receptacles treated with these compounds raises considerable concerns within the area of human health, given the facile migration of these substances into consumables, thereby resulting in elevated PFAS levels in human consumption [7]. The multifaceted interaction of PFAS with environmental matrices and the consequential implications for human exposure underscore the imperative nature of comprehensive investigations into the ecological and health impacts of these persistent synthetic compounds.

Two types of PFAS, specifically perfluorooctanoic acid (PFOA) and perfluorooctane sulfonate (PFOS), are of particular concern. Notably, these substances exhibit prolonged half-lives of four to five years in the human physiological milieu, coupled with the capacity to traverse the placental barrier, thereby instigating bioaccumulation processes [7]. The presence of PFAS in serum poses multifaceted implications for various physiological systems within diverse health conditions. Their impact encompasses the modulation of thyroid, kidney, liver, lung, and immune functions, as well as lipid metabolism, thereby elevating the risk of metabolic syndrome [8]. PFAS are also identified in the serum of individuals afflicted by anxiety and depression [9]. In fact, the current body of literature regarding the potential correlation between PFAS exposure and anxiety/depression-like behavior is still inconclusive and lacks consistency.

However, experimental evidence does suggest that there may be an impact on anxiety-like behavior resulting from exposure to PFAS [10] by interfering with the hypothalamic-pituitary-adrenal (HPA) axis [11,12]. Exposure to PFAS increases the risk of developing or worsening mental health disorders due to their neurotoxic effects [13,14,15]. Experimentally, these findings were also modeled specifically in PFOA-exposed mice, which exhibited anxiety-like behavior and increased corticotrophin-releasing hormone (CRH) expression in the basolateral amygdala complex (BLA), which have been found to participate in the pathogenesis of several psychiatric disorders [16]. In humans, prenatal PFAS exposure seems to be a significant risk factor for depressive symptoms in pregnant women [17], along with higher BASC-2 (Behavioral Assessment System for Children-2) scores for externalizing problems, hyperactivity, aggression, and conduct problems [18].

Depressive disorder stands as a significant contributor to the global burden of disability-adjusted life years, with over 300 million individuals affected in 2015, constituting 4.4% of the world’s population [19,20]. In Europe, approximately 31 million adults suffer from depression [21]. However, there is a lack of research on the possible negative impact of PFAS on mental health. Thus, our goal is to examine the association between plasma PFAS levels and depression prevalence in a representative population-based sample of adults and older adults. Given the recognized role of PFAS as endocrine disruptors, with a plausible influence on adipose tissue mass [22,23], and the established interplay between depression and obesity, notably through the hyperactivation of the hypothalamic-pituitary-adrenal (HPA) axis [24,25], our inquiry extends to elucidate potential connections with a focus on body fat status. The intricate relationship between depression and obesity is particularly relevant in the context of stress-induced mechanisms [26]. Thus, we pay particular attention to the associations adjusted and stratified by body fat status.

## 2. Materials and Methods

### 2.1. Study Design and Population

The participants in this study are part of a probability population-based sample, which is representative of the Kardiovize study, an epidemiological cohort comprising 2430 individuals aged between 25 and 89. The study randomly selected 479 individuals, consisting of 279 from the youngest cohort and 200 aged 65 or older. Ethical considerations adhered to the principles outlined in the Helsinki Declaration, ensuring the protection of participant rights and well-being. All individuals involved in the study provided explicit and informed consent before participation. The study protocol received approval from the ethics committee of St. Anne’s University Hospital in Brno, Czech Republic.

### 2.2. Demographic Variables

Health professionals affiliated with the International Clinical Research Center at St. Anne University Hospital in Brno employed the Research Electronic Data Capture (REDCap), a web-based electronic survey tool, to facilitate face-to-face interviews with patients. This methodological approach was chosen to gather comprehensive data on various facets of the participants’ demographic profile. The survey encompassed a spectrum of demographic variables, providing insights into crucial aspects of the participants’ backgrounds. These variables included age, educational attainment, and socioeconomic status, as well as habits related to smoking and alcohol consumption. The educational level was stratified into three categories: primary, secondary, or higher education. Concurrently, the socioeconomic status was delineated based on monthly household income, with categorizations of low (<EUR 1200), middle (EUR 1200–1800), or high (>EUR 1800). 

Smoking status was characterized as either “non-smokers” or “current smokers”, the latter encompassing individuals who reported smoking daily or less than daily over the past year. Participants’ alcohol consumption habits were dichotomized into “non-drinkers”, which included abstainers and those who had refrained from drinking in the previous 12 months, and “drinkers”, based on their reported alcohol intake over the last week, quantified in terms of standard drinks. A standard drink was standardized across various alcoholic beverages, equating to a glass of wine, a bottle of beer, or a shot of spirits, each containing approximately 10 g of ethanol.

Diabetes was operationally defined as either having a fasting blood glucose level equal to or exceeding 126 mg/dL (7.0 mmol/L) [27] or self-reporting a diagnosis of diabetes. Hypertension criteria included a systolic blood pressure of 140 mmHg or higher, a diastolic blood pressure of 90 mmHg or higher [27], a personal history of hypertension, or the use of antihypertensive medication. Waist circumference, a key anthropometric measure, was assessed midway between the bottom of the ribs and the top of the hips using a manual tape, providing an objective index of central adiposity. Physical activity was assessed through the Long version of the International Questionnaire of Physical Activity [28], where participants were considered “active” if they engaged in vigorous physical activity for three or more days per week, for at least 20 min per day, or moderate-intensity physical activity or walking for five or more days, for at least 30 min per day, or any combination of walking, moderate-intensity, or vigorous-intensity activities for five or more days per week, achieving a minimum of at least 600 metabolic equivalents for the task (MET)-min/week. Participants who did not perform any of the activities above were classified as “insufficiently active”.

### 2.3. Depression

To assess depression symptoms in those under the age of 65, we employed the Patient Health Questionnaire (PHQ-9) [29]. This clinical screening instrument comprises nine questions designed to align with the symptoms of depression as delineated in the Diagnostic and Statistical Manual of Mental Disorders (DSM-IV). The PHQ-9 generates a comprehensive score ranging from 0 to 27, with a designated depression cutoff score of ≥10. For individuals over 65, we utilized the validated Center for Epidemiologic Studies Depression scale (CES-D), which evaluates the number and duration of depressive symptoms through 10 questions. A standard cutoff score of ≥10 out of 30 possible points applies to the CES-D [30].

### 2.4. Measurement of PFAS Serum Concentrations

Samples of frozen blood serum underwent meticulous analysis to ascertain the presence of four PFAS compounds: perfluorooctanoic acid (PFOA), perfluorooctane sulfonate (PFOS), perfluorononanoic acid (PFNA), and perfluorodecanoic acid (PFDA). This analytical procedure was conducted at the RECETOX laboratory, a highly accredited trace analytical facility. The serum samples were subjected to a systematic processing sequence, initiated by allowing them to attain room temperature and subsequently homogenizing them using a vortex. Subsequently, 200 µL of each sample was meticulously transferred to a 96-well Phree Phospholipid Removal Plate (Phenomenex, Torrance, CA, USA), followed by filtration and relocation to glass vials. The samples were then evaporated under a nitrogen stream until the last drop of solvent was obtained. Finally, 50 µL of methanol and 50 µL of NH4Ac in water were added before the samples underwent analysis utilizing an LC-MS/MS system—specifically, the LC Agilent 1290 connected with QTrap 5500 (ABSciex, Framingham, MA, USA). The chromatographic separation was facilitated by a SYNERGI 4 µ Fusion MAX-RP 80 Å 100 mm × 2 mm column (Phenomenex, Torrance, CA, USA), with a pre-column Phenomenex SecurityGuard C18 4 × 2 mm. The analytical performance was carefully gauged, with the limit of quantitation (LOQ) for each compound set at 3 standard deviations (SD) of blank values and the limit of detection (LOD) at 1.5 SD. All four compounds (PFOA, PFOS, PFNA, and PFDA) were successfully detected in all samples using this robust methodology. Specifically, PFOA was found at a LOD of 0.020 ng/mL and a LOQ of 0.070 ng/mL, PFOS at a LOD = 0.030 ng/mL and a LOQ = 0.090 ng/mL, PFNA at a LOD = 0.004 ng/mL and a LOQ = 0.012 ng/mL, and PFDA at a LOD = 0.004 ng/mL and a LOQ = 0.010 ng/mL. The trace amounts detected underscore the sensitivity and precision of the analytical approach employed in elucidating the presence of these PFAS compounds in the blood serum samples.

### 2.5. Body Composition

To assess weight and body composition, an InBody 370 scale equipped with multi-frequency bioelectrical impedance analysis (BIA) and an eight-point tactile electrode system (BIOSPACE Co., Ltd., Seoul, Republic of Korea) was employed. It utilizes a direct segmental multi-frequency technique predicated on the conceptualization of the human body as five interconnected cylinders. The device leverages direct impedance measurements to delineate various body compartments [31]. Operating at multiple frequencies (5, 50, and 250 kHz), the BIA technique provides a nuanced analysis of the body’s composition. By assessing the spectrum of electrical frequencies, the technique extrapolates critical parameters such as the phase angle [32], intracellular water (ICW), and extracellular water (ECW) compartments within the total body water (TBW) across distinct body segments. Specifically, low frequencies are utilized to gauge the conductive properties of extracellular fluid, while high frequencies encompass the conductive properties of both intracellular and extracellular fluids. Lean body mass is derived by summing the intracellular and extracellular fluids and dividing the total by 0.73 [31]. The ensuing difference between total body weight and lean body mass yields the fat mass component. Moreover, the InBody 370 features an auto-calibration mechanism that activates each time the device is powered on, enhancing its reliability. This analytical method can function as a viable alternative to dual-energy X-ray absorptiometry (DXA), especially when the latter is unavailable [33]. High body fat cutoff points were established at 25% for men and 35% for women [34].

### 2.6. Statistical Analysis

In this study, a diverse array of methods was employed to represent both continuous and categorical variables. Continuous variables were characterized using the median and interquartile range, while categorical variables were presented as proportions in percentages. To enhance the normality of PFAS concentrations, a natural logarithm (ln) transformation was applied prior to analysis.

In addition to calculating the geometric mean, median, minimum, and maximum values of the transformed PFAS concentrations, the study utilized an adjusted Poisson regression model with robust error variances. This model was employed to assess prevalence rates (PR) concerning the association between continuous PFAS levels and dichotomous variables (presence/absence of depression). The utilization of the adjusted Poisson regression model facilitated the estimation of precise 95% confidence intervals (CI). To identify confounding variables, the researchers consulted existing literature [35] and systematically evaluated the available variables. The variable “type of questionnaire” was incorporated as a confounder, given the utilization of two different questionnaires to classify depression status. A covariate examination was conducted, and the outcomes were presented as PR. All statistical analyses were executed using STATA software (version 14.0, StataCorp, College Station, TX, USA), with a predetermined statistical significance level set at α ≤ 0.05. 

## 3. Results

A group of 467 individuals underwent examination, and the median age was found to be 52 years (with an interquartile range of 30). The female population accounted for 56.3% of the total group. Depression was found to affect 7.9% of the population, with a higher prevalence observed among women. Individuals who were classified as having “insufficient physical activity” had a higher prevalence of depression, while those who engaged in high physical activity reported fewer cases. These findings were consistent with the data presented in Table 1. The median percentage of body fat was 28.3% (with a standard deviation of 10.2), and high body fat was prevalent in 41.1% of the population.

In Table 2, the analysis of the four substances reveals that PFOS exhibited the highest concentration, with a median of 3.45 ng/mL, followed by PFOA with a median of 1.63 ng/mL, PFNA with a median of 0.58 ng/mL, and PFDA with a median of 0.18 ng/mL. These values provide a quantitative overview of the distribution of these PFAS compounds within the studied population. Table 3 presents the prevalence ratio (PR) values derived from the associations between different PFAS concentrations and the occurrence of depression. The results are presented for the entire sample and are adjusted for high adiposity (adiposity status), as well as the unadjusted values. 

The study’s findings present a significant association between PFOA and depression, both before and after adjusting for adiposity status. The prevalence ratio (PR) for PFOA was 1.97 (CI95%: 1.06–3.69) and 1.95 (CI95%: 1.04–3.65), respectively. While PFNA and PFDA exhibited positive associations with depression, these were not statistically significant. PFOS did not demonstrate any significant association with depression. Further exploration revealed that the significant associations between PFOA, PFNA, and PFDA and depression were observed exclusively in the normal adiposity group. For this subgroup, the PR values for PFOA, PFNA, and PFDA were 3.11 (CI95%: 1.53–6.34), 2.54 (CI95%: 1.32–4.90), and 2.10 (CI95%: 1.09–4.01), respectively. However, in the high-adiposity group, after additional analysis, it was observed that only physical activity level and body fat percentage retained statistical significance in relation to depression. Within this group, PFOA, PFNA, PFDA, and PFOS all exhibited a negative association with depression, with PR values of 0.43 (CI95%: 0.21–0.87), 0.43 (CI95%: 0.21–0.88), 0.44 (CI95%: 0.21–0.92), and 0.44 (CI95%: 0.21–0.91), respectively. Conversely, body fat percentage displayed a positive association with depression for all four variables, with PR values of 1.13 (CI95%: 1.04–1.22), 1.12 (CI95%: 1.04–1.21), 1.12 (CI95%: 1.04–1.21), and 1.11 (CI95%: 1.03–1.20), respectively.

## 4. Discussion

This study aimed to investigate the association between four PFAS compounds and depression, delving deep into the details of the relationship between these variables. The study’s findings revealed that among the four PFAS compounds analyzed, PFOA is most likely associated with depression, even after adjusting for adiposity status. The study found a significant association between PFOA and depression. While PFNA and PFDA showed positive associations, they were not found to be statistically significant. In contrast, PFOS did not exhibit any positive association with depression. These results suggest the importance of body fat status in the relationship between PFOA, PFNA, and PFDA with depression, with the strongest associations observed in individuals with normal adiposity. It is worth noting that the concentrations of PFAS found in the blood serum were lower than what is typically found in the literature, but they were consistent with the levels reported by the Czech national monitoring test [36]. In fact, although a cutoff for adverse health effects related to PFAS exposure is 2 nanograms per milliliter (ng/mL) of the sum of PFAS, there may not even be a level of PFAS exposure without some biological effect [37].

Disruption of neurotransmitters and neuromodulators by PFAS may contribute to an increased risk of neuropsychiatric disorders [10]. However, there are conflicting results in the literature regarding the association between depressive symptoms and serum levels of PFOS, PFOA, PFNA, and PFDA, among other PFAS. In a cross-sectional study by Berk et al. [38], which included 5400 subjects aged 18 years and older, unexpected associations were found between a lower risk of depression and higher levels of PFNA and PFDA. The study only considered the differences between the first and fourth quartiles of PFAS without adjusting or stratifying any variable related to body mass. In another cross-sectional study examining the relationship between PFAS exposure and CRH levels in mid-gestation according to different psychosocial stressors, the association between PFNA and CRH was stronger among women who experienced depression [39]. In older adults (55–74 years), no association was observed between depression and anxiety scores and PFOA [40]. Recently, PFOS was associated with higher depressive symptoms among immigrant women during pregnancy [17]. This highlights the importance of addressing the combined impact of PFAS exposure and social stress on health outcomes in marginalized communities [17]. 

Comparing studies is a challenge due to the varying characteristics of the sample, which is primarily composed of pregnant women [17,39]. However, in general terms, different PFAS seem to influence neurological health, confirming their action as endocrine disruptors. In some studies, neurotoxicity is linked to delayed gross motor development in infancy [41] and attention deficit hyperactivity in adolescents [42]. PFAS has been reported to exert significant toxic effects on HPA axis activity and on the dopaminergic system in several limbic brain regions [12,43], modifying the gene and protein expression of the glucocorticoid receptor (Gr) and the signaling of the brain-derived neurotrophic factor (Bdnf) [44]. Then, an association is suggested between PFAS and the activation of Gr signaling due to their direct Gr binding [45] and Bdnf signaling/HPA axis alterations in stress-related disorders, including depression [46]. Particularly, PFOA seems to activate the peroxisome proliferator-activated receptor α (PPARα) [47], which increases the hepatic expression of fibroblast growth factor 21 (FGF21) [48]. FGF21 stimulates the expression of the hormone CRH, and the increased concentration of CRH contributes to the etiology of disorders such as anorexia nervosa, obsessive-compulsive disorder, anxiety, and depression [48]. These observations suggest that some PFAS may act centrally to influence psychiatric disorders and eating behavior. The anorexic effect of PFAS also involves the activation of hypothalamic urocortin-2, CRH receptor-2, and suppression of gastroduodenal motor activity [49]. After stratifying the sample in the present study, these associations became apparent. An adjustment in the measure referring to adiposity amount removed potential confounding but blocked its mediating effect, and this was more evident for the PFNA and PFDA. 

The relationship between high adiposity and depression is complex and requires further exploration. Among individuals with high adiposity, physical activity levels and body fat percentage were found to be the most important factors associated with depression. It was only possible to confirm the association between body fat percentage and depression in this group, which is consistent with previous research [50,51]. Also, a few studies conceptualized the body mass index (BMI) as a proxy for dietary and physical behavior characteristics before pregnancy [52,53]. That would generate a potential “collider bias” when there are other causes common to adiposity levels [54]. In the current research, there was an attempt to minimize this by also adjusting the physical activity level. It can be reinforced by the fact that there was an inverse association between depression and physical activity level only in the group with high adiposity.

It is important to note that the current study has certain limitations that must be acknowledged. Specifically, no consideration was given to comorbidities such as neurodevelopmental and behavioral disorders or a prior history of mental illness. Also, although there are differences in serum PFAS levels among races in the general population [55], this variable was not collected. It is important to note that the Czech population is largely homogenous, with only approximately 5.3% of the population being made up of foreigners (primarily Ukrainians, Slovaks, Vietnamese, and Russians), and the ethnic minority Roma comprising just 2.2% of the overall population [56]. 

It is worth mentioning that the sample size was relatively small, and, as the study was cross-sectional, we cannot determine causation. Thus, further research, such as a longitudinal study, is required to confirm our findings. The previous studies’ mixed results indicate a need for diverse approaches when investigating the link between PFAS and mental health-related factors. A notable strength of this study is the inclusion of a sample that was not highly exposed to PFAS.

## 5. Conclusions

The present analysis suggests a distinctive association between PFAS levels in the blood and the prevalence of depression. Remarkably, among the four analyzed PFAS compounds, perfluorooctanoic acid (PFOA) emerged as the sole significant association before adjustments for adiposity levels. However, subsequent stratified analyses revealed that PFOA, along with perfluorononanoic acid (PFNA) and perfluorodecanoic acid (PFDA), demonstrated associations exclusively in individuals with a normal amount of adiposity. To our knowledge, this study is the first to establish a connection between PFAS levels and depression that is contingent upon body fat status. The identification of these associations highlights the need for further investigations to unravel the intricate mechanisms underlying the impact of PFAS on mental health. Depression remains a pervasive global concern, and elucidating the environmental factors contributing to its prevalence is of paramount importance. Moreover, these findings underscore the significance of considering individual factors, such as body composition, when evaluating the mental health consequences of environmental pollutants. This study contributes to a growing body of evidence suggesting that the impact of environmental pollutants on mental health outcomes is complex and multifaceted. As PFAS continue to be ubiquitous in contemporary life, understanding the interplay between PFAS exposure, body fat status, and mental health is crucial for comprehensive risk assessment and public health interventions.

## Figures and Tables

**Table 1:** Characteristics of the subjects in accordance with depression status (n = 467).

Variables	Depression (n = 37)	No Depression (n = 430)	*p*
Age	49 (32)	52 (30)	0.50
Women	73.0%	54.9%	0.03
Educational Level (%)			
Low	0.0%	3.5%	
Middle	59.%	56.3%	
High	40.5%	40.2%	0.76
Household Income (EUR) (%)			
Low (<1200)	64.9%	53.9%	
Middle (1200–1800)	13.5%	22.3%	
High (>1800)	21.6%	23.7%	0.36
Smoking (%)	16.2%	16.5%	0.96
Alcohol (%)	86.5%	90.2%	0.47
Physical Activity Level			
Insufficient	32.4%	13.7%	
Moderate	37.8%	38.4%	
High	29.7%	47.9%	0.001
Body Fat Percentage	31.0 (22.2)	27.7 (16.3)	0.19
Waist Circumference (cm)	90 (24.5)	92.5 (21)	0.33
High Body Fat (%)	35.0%	25.5%	0.96
Diabetes (%)	21.6%	14.9%	0.28
Hypertension (%)	43.2%	37.9%	0.52

Age, body fat percentage, and waist circumference presented as median (interquartile).

**Table 2:** Descriptive statistics of perfluoroalkyl (PFAS) variables (ng/mL).

	Geometric Mean	Median	Minimum	Maximum
Perfluorooctanoic acid (PFOA)	1.58	1.63	0.26	6.72
Pefluorooctane sulfonate (PFOS)	3.50	3.45	0.68	128
Perfluorononanoic acid (PFNA)	0.58	0.58	0.14	3.43
Perfluorodecanoic acid (PFDA)	0.17	0.18	0.01	1.34

**Table 3:** Associations between serum PFAS levels (ng/mL) and depression prevalence unstratified and stratified by body fat status.

PFAS	Entire Sample ^a^ PR (CI 95%)	Entire Sample ^b^ PR (CI 95%)	Normal Adiposity ^c^ PR (CI 95%)	High Adiposity ^c^ PR (CI 95%)
PFOA	1.92 (1.01–3.69)	1.91 (1.01–3.65)	3.20 (1.46–7.01)	0.78 (0.27–2.22)
PFOS	0.99 (0.56–1.76)	1.00 (0.56–1.79)	1.59 (0.76–3.30)	0.64 (0.26–1.58)
PFNA	1.73 (0.95–3.14)	1.74 (0.96–3.18)	2.54 (1.29–5.00)	0.84 (0.30–2.34)
PFDA	1.37 (0.84–2.23)	1.39 (0.85–2.27)	2.09 (1.09–4.00)	0.96 (0.50–1.82)

^a^—adjusted by age, sex, educational level, household income, smoking, physical activity level, diabetes, hypertension, waist circumference, and type of questionnaire used to classify depression status; ^b^—adjusted by age, sex, educational level, household income, smoking, alcohol, physical activity level, diabetes, hypertension, waist circumference, adiposity status, and type of questionnaire used to classify depression status; ^c^—adjusted by age, sex, educational level, household income, smoking, alcohol, physical activity level, body fat percentage, diabetes, hypertension, waist circumference, and type of questionnaire used to classify depression status; PR—prevalence rate.

## Data Availability

The data presented in this study are available upon request from the corresponding author. The data are not publicly available.
